# Supplementary material for: MK2a inhibitor CMPD1 abrogates chikungunya virus infection by modulating actin remodeling pathway
Source: PLoS Pathog. 2021 Nov 15;17(11):e1009667. doi: 10.1371/journal.ppat.1009667 (PMC8592423; doi:10.1371/journal.ppat.1009667)
Supplement: S1 Data — Table A. Differently modulated host genes for CHIKV-IS classified in to different metabolic pathways. Table B. Bioavailability prediction of CMPD1 through the SWISSADME web tool. Table C. Disease scoring of CHIKV infected and drug treated mice. Fig A. Effect of CMPD1 on CHIKV viral titer in HEK 293T cells. Fig B. TEM image showing CHIKV particles trapped inside Vero cells during CMPD1 treatment. (DOCX) [file ppat.1009667.s001.docx]

**Supporting information**

**S1 Data**

**Table A: Differently modulated host genes for CHIKV-IS classified intodifferentmetabolic pathways.**

| **Sl.no** | **Pathway** | **Entrez Gene ID** |
| --- | --- | --- |
| 1 | **Apoptosis signalling pathway** | LTA, EIF2S1, IKBKB. |
| 2 | **B cell activation** | RAC2, IKBKB. |
| 3 | **Cadherin signalling** | ACTC1, PCDH7. |
| 4 | **Cytoskeletal regulation by Rho GTPase** | RAC2, ACTC1. |
| 5 | **EGF receptor signalling** | RAC2, BTC, RASAL2, SHC1 |
| 6 | **Inflammation mediated by chemokine and cytokine signalling pathway.** | PLCB2, SOCS6, COL12A1, SHC1, ACTC1, IKBKB, IFNG. |
| 7 | **Integrin signalling pathway** | COL4A2, LIMS2, RAC2, COL12A1, COL4A4, FLNA, SHC1. |
| 8 | **Interleukin signalling pathway** | SHC1, SPIC. |
| 9 | **Notch signalling** | LNX1, LOC100428736. |
| 10 | **Oxidative stress.** | DDIT3, MEF2C. |
| 11 | **Ras pathway** | RAC2, MAPKAPK3, SHC1. |
| 12 | **PDGF signalling** | SHC1, IKBKB, SRGAP1. |
| 13 | **P38 MAPK signalling** | RAC2, **MAPKAPK3**, MEF2C. |
| 14 | **T cell activation** | RAC2, LCP2, IKBKB. |
| 15 | **TGF beta signalling pathway** | TGFB3, SMAD5, BMP1, SMURF1. |
| 16 | **Ubiquitin proteasome** | UBE2A, SMURF1. |
| 17 | **Wntsignalling** | PLCB2, PCDH7, SMAD5, ACTC1, DCHS1. |

**Table B: Bioavailability prediction of CMPD1 through the SWISSADME web tool.**

| Sl.No | **PHYSICOCHEMICAL PROPERTIES** | |
| --- | --- | --- |
| 1 | Formula | C22H20FNO2 |
| 2 | MW | 349.4 |
| 3 | #Heavy atoms | 26 |
| 4 | #Aromatic heavy atoms | 18 |
| 5 | Fraction Csp3 | 0.14 |
| 6 | #Rotatable bonds | 7 |
| 7 | #H-bond acceptors | 3 |
| 8 | #H-bond donors | 2 |
| 9 | MR | 102.27 |
| 10 | TPSA | 49.33 |
|  | **LIPOPHILICITY** | |
| 1 | iLOGP | 3.11 |
| 2 | XLOGP3 | 4.72 |
| 3 | WLOGP | 5.39 |
| 4 | MLOGP | 4.43 |
| 5 | Silicos-IT Log P | 5.28 |
| 6 | Consensus Log P | 4.59 |
|  | **WATER SOLUBILITY** | |
| 1 | ESOL Log S | -5.03 |
| 2 | ESOL Solubility (mg/ml) | 3.26E-03 |
| 3 | ESOL Solubility (mol/l) | 9.33E-06 |
| 4 | ESOL Class | Moderately soluble |
| 5 | Ali Log S | -5.49 |
| 6 | Ali Solubility (mg/ml) | 1.14E-03 |
| 7 | Ali Solubility (mol/l) | 3.27E-06 |
| 8 | Ali Class | Moderately soluble |
| 9 | Silicos-IT LogSw | -8.31 |
| 10 | Silicos-IT Solubility (mg/ml) | 1.71E-06 |
| 11 | Silicos-IT Solubility (mol/l) | 4.89E-09 |
| 12 | Silicos-IT class | Poorly soluble |
|  | **PHARMACOKINETICS** | |
| 1 | **GI absorption** | **High** |
| 2 | BBB permeant | Yes |
| 3 | Pgp substrate | No |
| 4 | CYP1A2 inhibitor | Yes |
| 5 | CYP2C19 inhibitor | Yes |
| 6 | CYP2C9 inhibitor | Yes |
| 7 | CYP2D6 inhibitor | Yes |
| 8 | CYP3A4 inhibitor | Yes |
| 9 | log Kp (cm/s) | -5.08 |
|  | **DRUG LIKENESS** | |
| 1 | Lipinski #violations | 1 violation: MLOGP>4.15 |
| 2 | Ghose #violations | 0 |
| 3 | Veber #violations | 0 |
| 4 | Egan #violations | 0 |
| 5 | Muegge #violations | 0 |
| 6 | **Bioavailability Score** | **0.55** |
|  | **MEDICINAL CHEMISTRY** | |
| 1 | PAINS #alerts | 0 |
| 2 | Brenk #alerts | 1 alert: hydroquinone |
| 3 | Leadlikeness #violations | NO; 1 violation : XLOGP3>3.5 |
| 4 | Synthetic Accessibility | 2.14 |
|  |  |  |

**Table C: Disease scoring of CHIKV infected and drug treated mice**

| **NO OF DAYS** | **MOCK**  **(N=5)**  **No of mice (Disease score)** | **CHIKV IS Infected**  **(N=5)**  **No of mice (Disease score)** | **CHIKV IS Infected + CMPD1 treatment (5mg/kg) (N=5)**  **No of mice (Disease score)** |
| --- | --- | --- | --- |
| DAY 1 | 5- (0) | 5- (1) | 5- (1) |
| DAY 2 | 5- (0) | 5- (1,2) | 5- (1,2) |
| DAY 3 | 5- (0) | 5- (1,2) | 5- (1,2) |
| DAY 4 | 5- (0) | 3-(3), 2(1,2) | 5- (0) |
| DAY5 | 5- (0) | 1-(5), 2-(4), 2-(3) | 5- (0) |
| DAY 6 | 5- (0) | 1-(6), 2(5), 2-(4) | 5- (0) |
| DAY 7 | 5- (0) | 2-(6), 2-(5) | 5- (0) |
| DAY 8 | 5- (0) | 2-(6) | 5- (0) |

[(0- No symptoms, 1- lethargic, 2- ruffled fur, 3- restricted movement/limping, 4- one hind limb paralysis and 5 – both hind limb paralysis 6- Morbid/dead)]


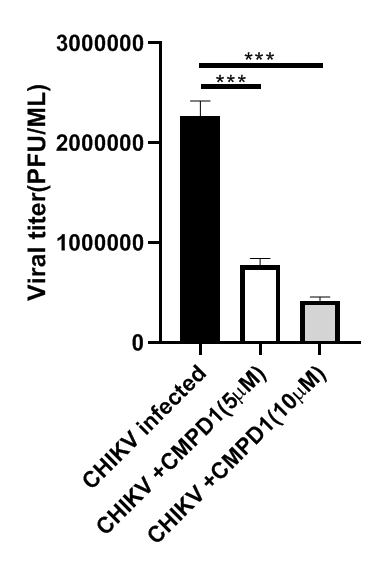


**Fig A: Effect of CMPD1 on CHIKV viral titer in HEK 293T cells**. HEK293T cells were infected with the IS strain (0.1 MOI), 5µM and 10µM of CMPD1 were added to the cells separately and incubated for 18 hpi. Bar graph showing the viral titers of CHIKV in HEK293T cell line (n=3;p≤0.05).

IS + CMPD1(50µM)

IS only

Mock

**
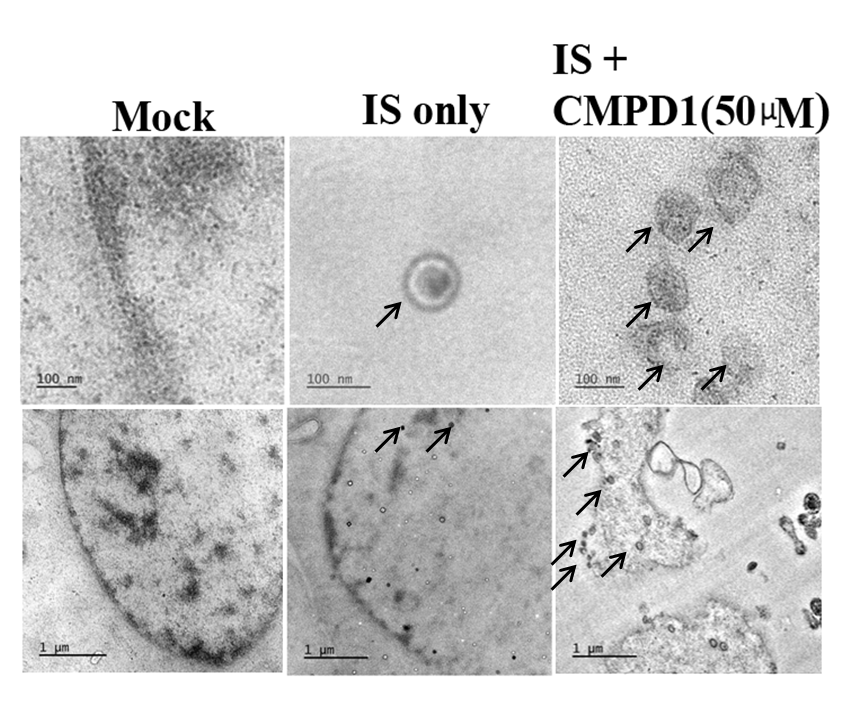
**

A

F

E

D

C

B

**Fig B: TEM image showing CHIKV particles trapped inside Vero cells during CMPD1 treatment.**Vero cells were CHIKV-infected, drug treated and were harvested at 18 hpi, fixed, dehydrated and embedded in epon resin. The ultra-thin sections (70 nm thick) were cut using a Leica EM UC7 ultramicrotome and processed for TEM imaging.**(A and B).** Uninfected cells.**(C and D)** High and low magnification overview of infected cells showing negatively stained mature CHIKV particles.**(E and F)**High and low magnification overview of CMPD1-treated virus infected Vero cells.
