# Supplementary material for: MK2a inhibitor CMPD1 abrogates chikungunya virus infection by modulating actin remodeling pathway
Source: PLoS Pathog. 2021 Nov 15;17(11):e1009667. doi: 10.1371/journal.ppat.1009667 (PMC8592423; doi:10.1371/journal.ppat.1009667)
Supplement: Attachment — Submitted filename: Rebuttal of Plos pathogens final 280921.docx [file ppat.1009667.s002.docx]

**REBUTTAL LETTER**

**First of all, we wish to thank all the reviewers for their critical evaluation of our manuscript and giving us valuable suggestions to strengthen our manuscript. We also wish to thank you for considering our manuscript to review and giving us opportunity to revise it based on the reviewers suggestions. We have tried our best to answer all the questions raised by reviewers point to point in our response letter as mentioned below and made the appropriate changes accordingly in our revised manuscript in track change mode.**

**Reviewer 1:**

1. I am not sure how microarray data has been analyzed. Author may take help from a professional bio informatics person. Author could use graph theory to analyze the micro array data. What is the rational to measure these cytokines as a pro-inflammatory one? Why not IL-1beta or TNFalpha, author should explain.

***Our Response*:** Thank you for the suggestion. We would like to inform you that the entire microarray raw data set has been analyzed by a professional bioinformatics person and thereafter submitted to the array express database, where it was first validated and then approved for submission. The reviewer user ID and password as well as the website link had already been submitted during manuscript submission.

The proteome profiler data was generated for samples of one time point. At that particular time point where samples were harvested, there was not much difference in the expression levels of TNF or IL-1β between the infected and drug treated samples. Hence, only those cytokines/chemokines have been included which had significant differences in their expression patterns at that time point.

2. Discussion part is too lengthy.

***Our Response*:** Thank you for the suggestion. Accordingly, the Discussion part has been reduced in the updated manuscript file.

**Reviewer 2 :**

1. The authors compared two strains of CHIKV - PS and IS. The rationale why any comparison is needed at all is unclear. More importantly, the comparison complicated downstream analyses. Specifically, the time needed to complete a single round of replication appears to be different in these two strains. Based on their previous publication (ref #21), the pfu of one strain peaked at 8 hours post infection whereas the other peaked only at 20 hours post infection. Moreover, at 20 hours post infection, the faster replicating strain showed significant decline in pfu at this time point. Moreover, more than 50% of the cells infected with the faster replicating strain have shown cytopathic effects (CPE) as early as 12 hours post infection. Consequently, the comparison of the mRNA transcript levels at 20 hours post infection (line 146) is not comparing apples with apples. One strain would have undergone 3 (possibly 4) rounds of infection whereas the other would have just completed a single round of replication. The quality of the mRNA when >50% of the cells show CPE would also be doubtful.

***Our Response* :**Thank you for the suggestion. We would like to inform you that the whole purpose of taking two strains for the study was to assess whether thefaster replicating strain(IS) modulates any unique host factors which is either not modulated or differently modulated by the prototype strain (PS) whichprobably had an impact for its faster replicating property. This has been clarified also in the revised text (line no 114 to119).

Next, to compare the host factor modulation in the same phase of replication between the strains, the microarray data for 8hpi of IS (fast replicating strain) was compared with the data of 18hpi of PS (slow replicating strain). Moreover, in order to analyze the host factors modulated during early phase of CHIKV replication, the 8hpitime point for PS was included in the study. The quality of mRNA was evaluated before the microarray experiment and it passed QC. Thus, we don’t anticipate any problem with the mRNA quality.

2. Related to point 1, the effects of MK2/MK3 silencing on PS and IS strain infection are also difficult to interpret without comparing equivalent rounds of replication.

***Our Response*:** Thank you for the suggestion. Though, the replication rates for both these strains are different yet the effect of MK2 and MK3 silencing was same for both the strains post CHIKV infection. There was no reduction in viral protein expression however the viral titers were significantly reduced for both the strains.(58% for PS and 53% for IS strain respectively). This indicates that these host factors are essential for efficient viral infection and in absence of them viral infection was abrogated irrespective of the replication efficiency.

3. The use of Vero cells without validation of the hits on a relevant human cell also complicates interpretation of the findings. This is particularly since the goal of this study is to identify a suitable target for therapeutic development. Ideally, the gene expression study should have been done in a relevant human cell line.

***Our Response*:** Thank you for the suggestion. We would like to inform that we have also assessed the effect of CMPD1 on CHIKV replication in Human Embryonic Kidney (HEK) 293T cell line. This has been incorporated in the revised manuscript as S1 Fig and it was found out that there was more than 80% decrease in viral titer post CHIKV infection even with 10µM of CMPD1. This shows that the host factors are important in human cells also for CHIKV infection. This data has been incorporated in the revised manuscript (Line no 338 to 341).

4. The rationale for focus on MK2 and MK3 is unclear. The data in Table S1 is inadequate. The authors should show the mean and SD of each of the genes that were listed in this table. More appropriately, the entire microarray dataset should be made available for scrutiny. Without detailed data, it would seem that the authors cherry picked MK2 and MK3 for further examination, especially since the pathways highlighted in Table S1 are not consistent with those shown in Figure 1B and C.

***Our Response*:** As mentioned in response 1, the entire raw data set has been scrutinized and then approved for submission in the array express database.The reviewer user ID and password as well as the website link had already been submitted during manuscript submission.

It has been reported earlier by us that P38MAPK is crucial for CHIKV infection (Nayak et al., *Front Immunol*, 2019), so we wanted to investigate the role of some downstream host factors of P38MAPK pathway which can be targeted for developing antivirals in future and would be less lethal to the host unlike targeting P38MAPK. Hence, MK2 and MK3 were selected for this study. We have mentioned the reason of working with these host factors in the revised manuscript in the (line no 99 to 111).However, there are few other host factors which are also being studied in our lab that would be submitted as a separate manuscript in future.

5. The notion that MK2 and MK3 silencing led to inhibition of "CHIKV progeny release" is not well supported by the data. The authors only measured nsP2 levels by western blot. On the other hand, CHIKV replication involves multiple steps, from virus uncoating to RNA transcription and translation, to viral protein folding, vision assembly and finally egress. The authors only showed that nsP2 could be detected and was marginally increased with MK2 and MK3 knockdown. The specific step in which virus infection/replication was inhibited by the reduction in MK2 and MK3 is entirely unclear without more detailed study. The data Figure 3F and G could be argued to support the notion that CHIKV was formed but could not egress the cell with CMPD1 treatment. However, this data is preliminary and the impact of non-MK2/MK3 dependent effects of CMPD1 on viral assembly/egress has not been excluded.

***Our Response*:** Thank you for this important suggestion. We would like to inform that in order to support our hypothesis, Transmission Electron Microscopy (TEM) imaging of the Vero cells have been carried out in presence and absence of CMPD1 post CHIKV infection and it is observed that more number of CHIKV particles were trapped inside CMPD1 treated CHIKV infected cells in comparison to untreated CHIKV infected cells as shown in S2 Fig. This finding along with the plaque assay data (Fig 3F and 3G) can support our hypothesis. This has also been incorporated in the revised text (Line no 237 to 247, 362 to 365).

6. The *in vitro* experiment with CMPD1 is problematic. The authors should show the actual MTT assay data. Conventionally, any statistically significant reduction in cell viability compared to mock treated control would not be used to assess for antiviral activity, as reduction in cell viability will reduce viral titers. The data described in line 323 is thus insufficient. Moreover, any less than 90% cell viability is likely to be significantly different compared to control cells. CMPD1 at 100 uM should thus not have been used to calculate the IC50. Moreover, it is also unclear why the authors chose to no report the data with 75 uM of CMPD1 (line 322).

***Our Response*:**.In this regard, we would like to inform that the data upto 80µM has been included and 100µM concentration of CMPD1 has been removed to calculate the IC_50_ value as shown in Figure 3D and the revised IC50 value was calculated to be 21.18µM and this has been incorporated in the revised manuscript (Line no 340 to 344).

7. Even with the use of data from 100uM CMPD1 treatment to estimate IC50, the value of nearly 34 µM is high, especially since significant level of toxicity is likely reached at 100 uM. The potential for any clinical translation is thus questionable.

***Our Response*:** Thank you for the suggestion. The explanation to this query has been given in the previous response. We also hypothesize that CMPD1 can be modified or derivatized to a more effective anti CHIKV therapeutic molecule in future.

8. The p38 pathway has been implicated in the pathogenesis of several other viral infections, including those discussed in this manuscript. The authors should thus have examined if some of these reported mechanisms, including control of RIPK1 signalling that is important for inflammation and infection, as reported by Menon et al (Nat Cell Biol 2017; 19:1248-59), could explain the observed outcome with CMPD1 treatment. Without excluding such known antiviral and anti-inflammatory pathways, the mechanism of action offered by the authors in Figure 7 is premature.

***Our Response*:** Thank you for the suggestion. The following point has been addressed.There was a significant increase in pRIPK1 in presence of CHIKV infection and this was decreased during CMPD1 treatment. We are facing difficulty to connect this part in the manuscript, hence this figure has been given for the reviewers only in the supplementary information 2 (S3 Fig). Please suggest further.

9. Line 46. "effective" should be changed to "licensed".
   Line 97. CHIKV is not conventionally described as small.
   Line 292. What is "antagonically"?

***Our Response*:** Thank you for the suggestion. The above mentioned points have been addressed in the revised manuscript file.

**Reviewer 3 :**

1. Authors designed elaborate study and performed many experiments, which are described in detail. However, Materials and methods section, and Results section are too lengthy. In addition, it seems abrupt to add experiments with SHV and SARS-CoV-2. Readers may feel it as in a hit-or-miss manner, because of the finding with CHIKV. Therefore, authors should considerably shorten the manuscript as much as possible, and consider to delete the part of SHV and SARS-CoV-2, which may be published as other report.

***Our Response*:** Thank you for the suggestion. As per the suggestion, the Material and methods and the Results section have been reduced in the revised manuscript file. Additionally, the experiments with HSV and SARS CoV2 have been deleted and will be published as a new report in future.

2. Authors identified MK2/MK3 as host factors, and their inhibitor CMPD1 was used for further analysis. However, there is no explanation of them in Abstract, and names of them did not appear in Introduction. Readers who are not familiar with them may not understand this study, which is not appropriate as scientific article. If word limit is permitted, any brief explanation of MK2/3 and CMPD1 should be added to Abstract and Introduction. For example, MK2 as MAPK-activated protein kinase 2; MK2 is a checkpoint kinase involved in the DNA damage response, etc. Line 435-436, "MK2 and MK3 belonging to P38MAPK pathway" needs some more annotations.

***Our Response*:** Thank you for the suggestion. This point has been addressed and the modifications in the Abstract (Line no 32 to 35) and the Introduction section (Line no 99 to 111) have been updated accordingly in the revised manuscript.
